# Supplementary material for: Scale Up and Validation of Novel Tri-Bore PVDF Hollow Fiber Membranes for Membrane Distillation Application in Desalination and Industrial Wastewater Recycling
Source: Membranes (Basel). 2022 May 30;12(6):573. doi: 10.3390/membranes12060573 (PMC9229717; doi:10.3390/membranes12060573)
Supplement: Supplementary file 1 [file membranes-12-00573-s001.zip › membranes-1739267-supplementary.pdf]

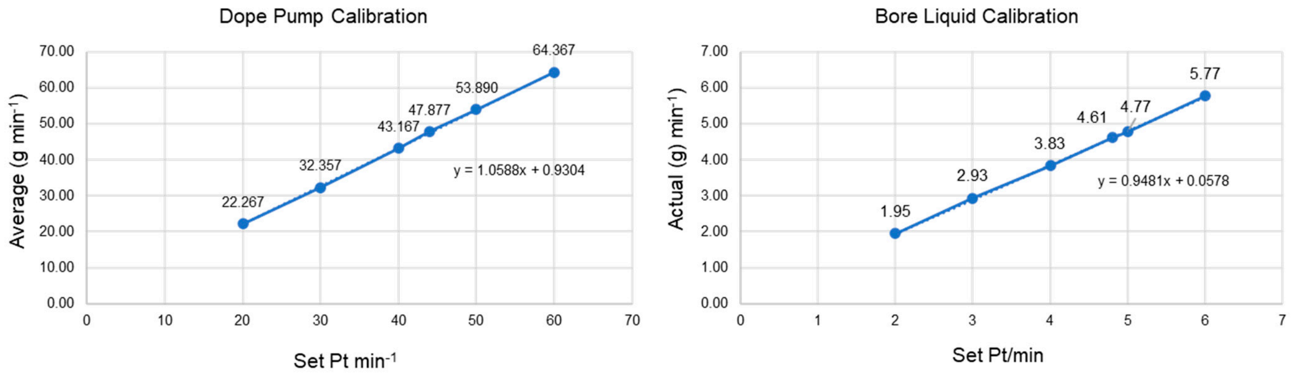

Figure S1. The calibration of the (a) dope pump and (b) bore liquid pump, as part of the membrane fabrication scale up process.

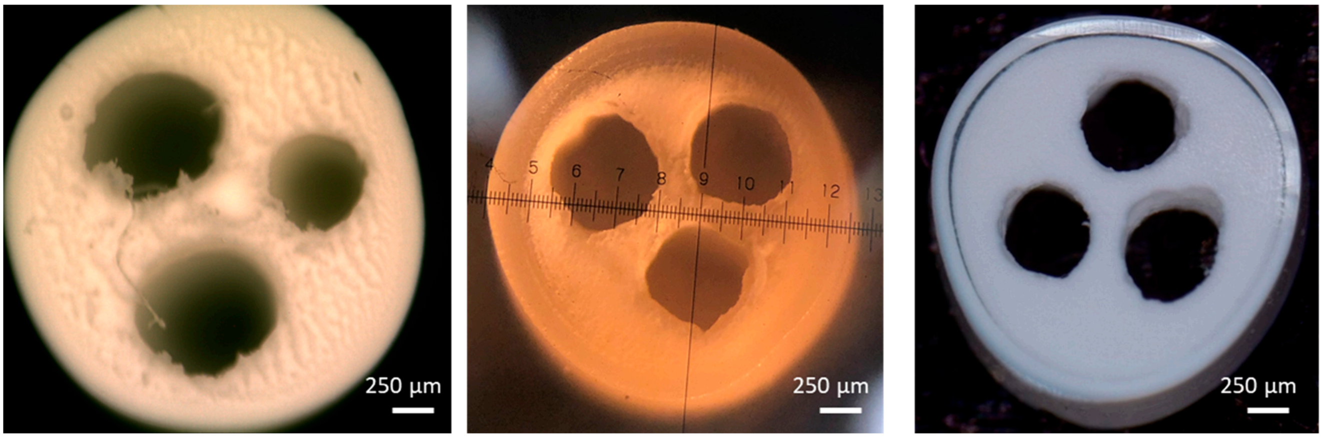

Figure S2. The optical microscope images used for determining the outer diameter (OD) and inner diameter (ID) of the tri-bore hollow fiber membranes.

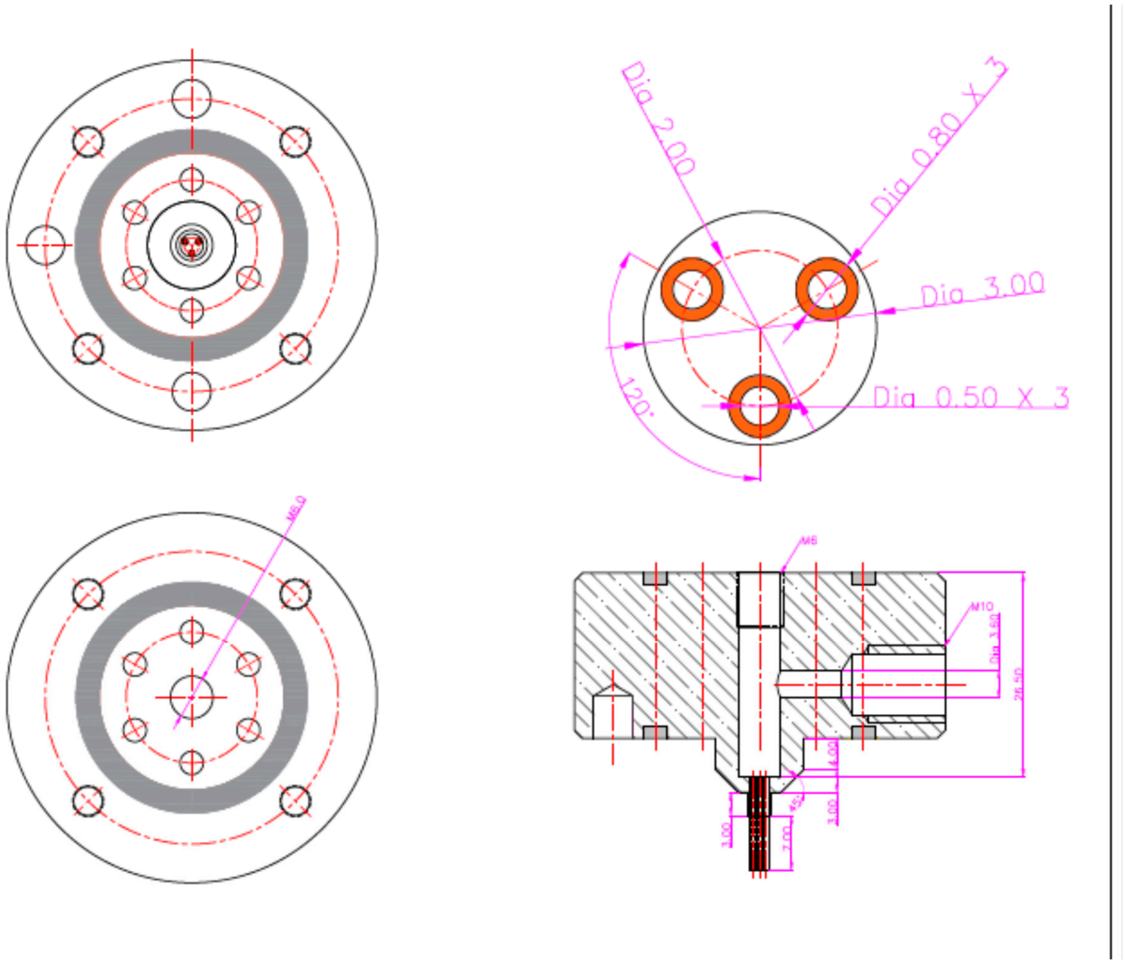

Figure S3. Spinneret design used for fabricating the tri-bore hollowfiber membrane.

Table S1. Porosity (%), contact angle (°), DCMD flux ($L\ m^{-2}\ h^{-1}$) and liquid entry pressure, LEP_w (bar) of different batches of 1.5 kg size.

Batch No	Batch Size	Porosity (%)	Contact angle (°)	LEPw (Bar)	DCMD Flux ($L\ m^{-2}\ h^{-1}$)	Rejection (%)
1	1.5 kg	72.8 ± 1.3	80.3 ± 3.1	2.5 ± 0.1	13.8 ± 0.3	>99.9
2	1.5 kg	72.4 ± 0.7	80.2 ± 9.8	2.6 ± 0.2	13.3 ± 0.3	>99.8
3	1.5 kg	75.4 ± 0.0	92.8 ± 0.7	2.6 ± 0.2	14 ± 0.3	>99.9
4	1.5 kg	74.5 ± 0.7	78.3 ± 3.5	2.6 ± 0.1	13.8 ± 0.3	>99.9
5	1.5 kg	69.0 ± 0.9	78.9 ± 8.1	2.7 ± 0.3	13 ± 0.5	>99.9
Average		72.8	82.1	2.6	13.6	99.8
Standard Deviation		2.5	6.0	0.1	0.4	0.1

Table S2. Raw data for DCMD optimization trials for PVDF TBHF membranes.

Module No	Test Mode	Membrane area (m^2)	Hot Flowrate (ml min^{-1})	Cold flowrate (ml min^{-1})	T hot in (°C)	T hot out (°C)	T Cold in (°C)	T Cold out (°C)	Flux (L m^{-2} h^{-1})
SN-1	DCMD (Out-to-In)	0.7	2000	500	81.9	62.6	11.1	75.9	1.92
SN-1	DCMD (Out-to-In)	0.7	2000	500	78.7	61.1	11.9	72.6	1.17
SN-2	DCMD (In-to-Out)	0.7	3000	1500	80.5	63.4	22.8	59.9	1.34
SN-2	DCMD (Out-to-In)	0.7	3000	1500	80.0	63.6	22.5	56.5	1.23
SN-3	DCMD (Out-to-In)	0.38	2000	1500	80.3	66.8	23.6	52.5	2.03
SN-3	DCMD (Out-to-In)	0.38	2000	1500	80.8	67.5	24.2	52.6	1.96
SN-3	DCMD (Out-to-In)	0.38	2000	500	81.6	73.6	11.6	66.7	2.78
SN-3	DCMD (Out-to-In)	0.38	2000	700	81.3	72.2	12.9	55.1	2.65
SN-5	DCMD (Out-to-In)	0.26	2000	500	80.1	76.2	14.2	55.6	4.80
SN-5	DCMD (Out-to-In)	0.26	2000	500	80.9	76.9	13.4	54.9	4.83
SN-5	DCMD (Out-to-In)	0.26	2000	700	80.6	74.8	13.5	46.0	4.69
SN-5	DCMD (Out-to-In)	0.26	2000	700	80.8	74.6	13.6	46.1	4.64
SN-5	DCMD (Out-to-In)	0.26	2000	500	81.9	76.8	13.6	55.1	4.63
SN-5	DCMD (Out-to-In)	0.26	2000	1000	80.9	74.4	13.3	40.4	4.80
SN-5	DCMD (Out-to-In)	0.26	2000	1000	80.9	74.8	15.5	41.7	4.68
SN-6	DCMD (Out-to-In)	0.26	2000	500	81.1	69.4	13.7	52.0	2.81
SN-6	DCMD (Out-to-In)	0.31	2000	700	79.9	66.9	15.5	43.5	2.69
SN-6	DCMD (Out-to-In)	0.31	2000	700	81.1	67.3	14.0	42.7	2.65
SN-6	DCMD (Out-to-In)	0.31	2000	500	81.2	68.4	14.0	49.2	2.56
SN-6	DCMD (Out-to-In)	0.31	2000	500	80.6	68.2	11.6	48.6	2.46

Table S3. Raw data for the VMD optimization trials for PVDF TBHF membranes.

Module No	Test Mode	Membrane area (m^2)	Hot Flowrate (ml min^{-1})	T hot in (°C)	T hot out (°C)	T Vapor Out (°C)	Vacuum pressure (bar)	Flux (L m^{-2} h^{-1})
SN-2	VMD(Out-to-In)	0.7	3000	82.8	77.8	76.1	-0.4	1.11
SN-2	VMD(Out-to-In)	0.7	3000	83.5	77.5	78.4	-0.4	0.96
SN-2	VMD(Out-to-In)	0.7	3000	81.6	73.4	75.1	-0.35	1.21
SN-2	VMD(Out-to-In)	0.7	3000	81.8	63.5	78.0	-0.21	0.84
SN-2	VMD(Out-to-In)	0.7	3000	82.5	75.8	78.2	-0.4	0.11
SN-2	VMD(Out-to-In)	0.7	3000	83.1	78.9	83.2	-0.6	0.67
SN-2	VMD(Out-to-In)	0.7	3000	82.3	77.3	78.4	-0.7	1.13
SN-3	VMD(Out-to-In)	0.38	3000	81.4	81.1	72.4	-0.7	1.99
SN-3	VMD(Out-to-In)	0.38	3000	80.7	79.3	71.3	-0.7	3.83
SN-3	VMD(Out-to-In)	0.38	3000	81.6	80.1	73.1	-0.4	1.78
SN-3	VMD(Out-to-In)	0.38	3000	80.5	78.8	70.3	-0.4	2.20
SN-3	VMD(Out-to-In)	0.38	3000	80.8	79.4	70.6	-0.3	2.09
SN-3	VMD(Out-to-In)	0.38	3000	81.4	79.8	71.2	-0.7	2.04
SN-3	VMD(Out-to-In)	0.38	3000	81.8	80.2	70.9	-0.7	2.25
SN-3	VMD(Out-to-In)	0.38	3000	80.9	79.4	69.5	-0.7	2.35
SN-3	VMD(Out-to-In)	0.38	3000	81.7	80.0	70.0	-0.7	2..18
SN-3	VMD(In-to-Out)	0.38	3000	80.4	74.7	71.6	-0.7	3.87
SN-3	VMD(In-to-Out)	0.38	3000	81.1	75.2	71.8	-0.7	3.90
SN-2	VMD(In-to-Out)	0.7	3000	81.2	74.0	73.1	-0.7	2.48
SN-2	VMD(In-to-Out)	0.7	3000	81.1	71.9	70.0	-0.7	3.59
SN-2	VMD(In-to-Out)	0.7	3000	80.9	72.1	70.4	-0.7	3.60
SN-2	VMD(In-to-Out)	0.7	3000	81.2	72.0	70.3	-0.7	3.48
SN-6	VMD(Out-to-in)	0.7	3000	80.6	76.6	70.8	-0.7	1.58
SN-6	VMD Double Outlet	0.31	3000	80.2	76.2	70.4	-0.7	1,84
SN-3	VMD Double Outlet	0.31	4000	80.9	76.0	71.7	-0.7	2.58
SN-3	VMD Double Outlet	0.38	5000	89.8	86.5	74.3	-0.7	4,75
SN-3	VMD Double Outlet	0.38	6000	90.4	86.5	74.5	-0.7	5.18
SN-3	VMD Double Outlet	0.38	6000	94.8	90.7	77.2	-0.7	6.21
SN-3	VMD Double Outlet	0.38	6000	89.9	85.2	69.6	-0.76	5.91
SN-3	VMD Double Outlet	0.38	6000	90.1	85.0	65.3	-081	6.97
SN-4	VMD(In-to-Out)	0.38	3000	81.1	73.6	68.3	-0.73	4.43
SN-4	VMD(In-to-Out)	0.38	3000	81.4	72.8	67.3	-0.74	5.04
SN-4	VMD(Out-to-In)	0.38	3000	80.4	78.2	72.4	-0.71	2.68
SN-4	VMD(Out-to-In)	0.38	3000	80.7	76.8	67.9	-0.75	2.66
